# Does Product Semantics Matter in Stimulating Impulse Buying Behavior for Internet Products?

**DOI:** 10.3389/fpsyg.2021.676086

**Published:** 2021-08-23

**Authors:** Xiangmeng Huang, Ruilin Cai

**Affiliations:** Business School, Changshu Institute of Technology, Suzhou, China

**Keywords:** design-led innovation, product semantic, internet product, anticipated regret, impulse buying

## Abstract

Design-driven innovation has become the source of the third-dimensional innovation driving force behind technology and outside the market, aiming to explore breakthrough innovation in product semantics for Internet products. This research tries to define the concept of product semantics and construct a consumer purchase decision model for Internet products with product semantic perception as the antecedent variable. In addition, how product semantics could stimulate consumers' expected regret and impulse purchase for Internet products is explained. The research finds that product semantic perception significantly affects consumers' expected inaction regret, which promotes their impulse purchase intention for Internet products; and expected inaction regret partially mediates between product semantic perception and impulse purchase intention. Self-control ability of consumers negatively moderates the relationship between their expected inaction regret and impulsive purchase intention for Internet products. Thus, the “non-use function” design of product semantics can effectively meet and lead the spiritual and cultural needs in hedonistic Internet shopping for consumers.

## Introduction

With the popularization of the Internet and other communication technologies, e-commerce has gained great popularity in China. According to a report by the Ministry of Commerce of China, China has become the world's largest e-commerce market, with its total revenue reaching 29.16 billion yuan in 2017 (ECCA, 2018). Over the past decade, it is notable that the online promotional activities launched by online platforms have led to shopping carnivals for Chinese consumers and further for the consumers worldwide, such as Taobao's annual “Double Eleven” festival. And not surprisingly, part of the explosion in sales at the yearly shopping festival comes from provisional impulse buying (Zhang et al., 2020). According to Danish Habib and Qayyum (2018), impulse buying for Internet products is common in online shopping through websites and social media platforms. It is claimed that more than 50% of all online purchases are made on impulse (Zheng et al., 2019; Wu et al., 2020). On the one hand, online businesses make every effort to develop promotion programs to induce consumers to purchase happily; on the other hand, consumers often struggle between regretful purchases and impulsive consumption, especially for Internet products which consumers could not physically touch immediately.

The research on impulse buying has aroused the attention of the academia and existing research mainly explores the related factors of the antecedent variables from the aspects of scenario and product. A variety of factors have been identified as predictors of impulse buying, including psychological factors (Rook and Gardner, 1993; Beatty and Ferrell, 1998; Amos et al., 2014; Bandyopadhyay, 2016), lack of control (Youn and Faber, 2000; Parboteeah et al., 2009; Amos et al., 2014), personality traits (Verplanken and Herabadi, 2001; Chan et al., 2017; Satyavani and Chalam, 2018), and demographic characteristics such as age (Wood, 1998), and gender (Dittmar et al., 1996; Silvera et al., 2008). It is widely believed that women are more likely to impulse buy than men as they are more likely to emphasize the emotional aspects of impulse buying. Moreover, it is also argued that besides pleasurable mood like excitement which could encourage impulse buying, consumers under negative mood states like sadness, were also likely to conduct impulse buying in order to improve their mood (Verplanken et al., 2005; Silvera et al., 2008).

Beyond these factors, other researchers have also found that the retailer environment, product mix design, and promotion design could also induce impulse buying (Zhou and Wong, 2004; Kacen et al., 2012; Mohan et al., 2013). However, little research has investigated how the culture and symbolism of the product influence impulse buying. With the increase of the degree of product homogenization, non-functional requirements are increasingly becoming the focus of customer attention. The online market is accelerating the shift of our society from a production-centered one to a consumption-centered postmodern society, which constitutes a cultural consumer society dominated and controlled by symbols such as patterns, images, and information. For example, when Kumar et al. (2015) studied brand preferences, they found that the cultural attributes of products can create emotional value for customers, thereby enhancing the brand's sense of dependence and loyalty; the reason is that product semantics create economic, functional, social, and emotional values for customers to meet consumer demand and lead consumer trends. Based on the theory of impulsive buying, this study explores the induction mechanism of product semantics to stimulate impulse purchases for Internet products on the basis of the cultural consumption needs of customers in postmodern society. It also collects primary data to test the relevant hypotheses through experimental research.

The contribution of this research could lie in two aspects. Firstly, the research on the impact of product semantics on online purchasing behaviors is helpful to reveal the mechanism of product semantics inducing anticipatory inaction regrets and impulse purchases, as well as enrich the purchasing decision theory. Secondly, it contributes to explore the relationship between product semantic symbol temptation and consumer impulse purchases, which has practical significance for promoting design-driven innovation.

## Literature Review

### Theoretical Basis

#### Impulse Buying Theory

Impulse buying is traditionally described as unplanned, compelling, and hedonically complex purchasing behavior (Stern, 1962). When exposed to provocative stimuli, consumers experience a sudden, strong, and persistent urge to purchase a specific item and they tend to make quick decisions with little or no attention paid to evaluating the item or to the consequences of such a purchase decision (Rook, 1987). Therefore, impulse buyers are apt to be unreflective in their thinking and emotionally attracted to an item, and their desires of immediate gratification frequently lead to dissatisfaction or regret after the purchase.

Products are the things that rely on their attributes to meet consumers' needs, thus the foundation for Basic Paradigm for Analyzing the Value of Commodities Based on Two Attributes of “Commodity-Goods” was established (Guo, 2014). In terms of the characteristics, product use functions are the basic requirements of consumers for purchasing goods, and product performance must be improved to avoid product quality loss (Pedersen and Howard, 2016). Thus, regret after impulse buying may be relieved. In addition to product characteristics, Flaherty and Mowen (2010) believed that consumption context is an important factor in purchasing behavior, that is, the specific transient background factors of consuming activities affect immediate purchasing decisions. Therefore, the two major aspects of product characteristics and consumption context have become the key influencing factors of consumer behavior, which also provide the basis for the “stimulus– organism-response” theory proposed by neo-behavioral psychology (Animesh, 2011). Besides product characteristics and situational factors, customers' perceptions and emotions of products are also stimulated and influenced by the consumption situation, which in turn induces impulse purchases. Recent years have seen attention aroused among scholars to the relationship between mixed marketing and impulse purchases. Previous research studies on impulse buying behaviors from the perspectives of product scarcity (Li et al., 2018), price discounts (Xu and Huang, 2014), combined promotions (Huang et al., 2018), terminal displays (Bai and Chen, 2016), community atmosphere (Chen et al., 2018), product knowledge level (Mohan et al., 2013), time pressure (Hao and Zeng, 2017), and so on, are thereby enriching the theory of impulse buying in the context of marketing stimulation. Though the psychological, environmental, situational, and mixed marketing factors have been discussed to induce impulse buying, the relationship between the product, such as culture expression, and semantic symbol temptation has not been established.

#### Mental Accounts Theory

Mental accounts theory is closely related to induced impulse buying. It is believed that people usually have two accounts, one is an economic account, and the other is a mental account. The economic account demonstrates that if a consumer categorizes a purchase as essential, they will define what they believe is a reasonable budget for a given category and within a given timeframe by considering their financial situation; while the mental account is for people who unconsciously assign resources to different accounts for management (Thaler, 1980). Furthermore, it is pointed out that the process of psychological operations under mental accounts is not the pursuit of maximizing the utility of rational cognition, but the maximization of emotional satisfaction, and there is a consumer experience under hedonism. Therefore, mental accounts often make consumers violate some simple economic principles when making decisions, and then make many irrational decisions or impulse purchases. Through the experimental research conducted by Seta et al. (2014), they found that the use of emotional care is the same as the use of money, which can also effectively adjust the consumer's evaluation and valuation of goods; so that the merchants can adjust the consumer's mental account perception rather than their economic account through spirit and culture. Liao (2014) summarized the studies of mental account theory and found that customers often violate simple algorithms and participate in irrational consumption due to bundling, price conversion, credit card payments, online shopping, expected losses, and so on, thus inducing impulse buying.

#### Impulse Buying for Internet Products

Impulse buying is generally thought of as a consumer behavior that is triggered by a sudden, powerful, and persistent impulse to purchase goods immediately (Rook and Fisher, 1995). The Internet has already been an integral part of daily life where customers can freely find information about products or services. Compared with traditional shopping, online shopping is more likely to lead to impulse buying. In particular, the feasibility of social media is a new approach that greatly facilitates marketing efforts and may play a key role in influencing consumer purchasing decisions, such as impulse buying (Alalwan et al., 2017; Kapoor et al., 2018). However, due to the complexity of the specific purchasing process, impulse buying for Internet products is still not well-understood, taking into account various viewpoints such as psychology and risk. Previous studies have proposed some analytical methods to find critical factors from social media-related information (Shiau et al., 2017; Stieglitz et al., 2018). It is believed that online impulsive buyers have higher emotional states and are more likely to bring about a spontaneous buying experience, and their shopping list is also not clear in advance (Fu et al., 2018). Thus, understanding why impulsive consumers purchase Internet products is especially important for online business purposes.

### Research Framework

Since impulse buying is profitable, online shopping businesses are committed to making consumers produce a higher perceived value of product semantics. The temptation symbol of “non-use functions” is used to stimulate consumers' impulse purchases. In addition, a higher product semantic perception is associated with the higher possibility of regret for losing the joy or excitement to buy the specific item, i.e., expected inaction regret.

Early research has addressed consumers' loss aversion which is prompting consumers to evaluate expected benefits and expected risks before buying. According to Janis and Mann (1977), the “loss” caused by the hypothetical benefits and risks before the purchase behavior of consumers was referred to as expected regret, that is, the “loss perception” generated by the results that consumers expected to give up before the purchase decision were better than the results they chose. Later on, Kanheman and Tversky (1982) further divided expected regret into expected action regret and expected inaction regret. The former refers to regret caused by consumers' expected purchase, while the latter refers to the regret felt by consumers when they give up the purchase. Both Camille et al. (2004) and Sandberg et al. (2016) argued that expected inaction regret is different from experience regret and it refers to the substantial “loss” perception and anxiety after the consumption experience. As previous literature has explored the relationship between expected action regret after purchase and impulse buying (Chang and Tseng, 2014; Lucas and Koff, 2014), this study focuses on the mechanism of impact on impulse purchases based on online consumer anticipatory inaction.

Marketing stimulus regulates the customer's mental account, and then induces customers to make online impulsive decisions for products. At the same time, the loss aversion caused by such impulse buying requires customers to take necessary self-control to reduce expected inaction and regret. There are three definitions of self-control: the first is the intuitive definition of the decision-maker's ability to resist temptation; the second is the axiomatic definition of exerting preference relation on the alternative set; the third is the definition of displaying preference (Jiang and Qu, 2016). Sharma et al. (2014) found that consumer impulse can be divided into three dimensions: cognitive flippancy, emotional indulgence, and lack of self-control behavior; self-indulgence and lack of self-control have no positive relationship with independent self-concept for consumers. Self-control can regulate impulsive behavior decision-making, but self-control is a kind of limited resource. Once used, the resources that individuals rely on for other self-control aspects will be reduced, which makes it difficult to reach the established performance standard of self-control and leads to the failure of regulating the individual's subsequent tasks (Dong and Ni, 2017). Studies have shown that self-control and long-term value orientation can help restrain consumption and increase willingness to thrift, but this effect is relatively obvious in the short term, while the long-term effect is not significant. Moreover, this effect is also regulated by the individual's material basis (Nepomuceno and Laroche, 2017). Self-control theory can explain why customers may not be able to resist the temptation of the marketing stimulus after they regret it and then make impulse purchases for the Internet product again.

**Figure 1:**
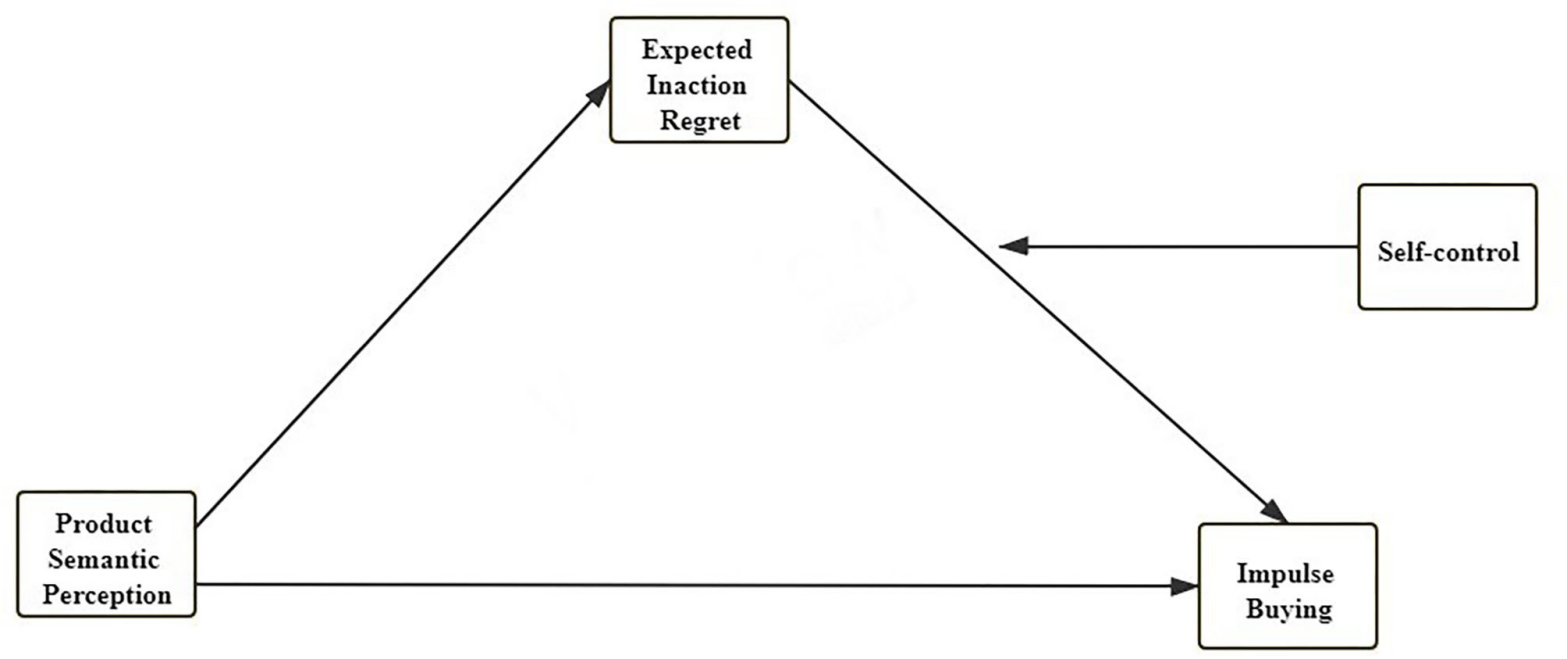
Conceptual model.

Thus, this paper attempts to explore purchasing behavior from a new perspective of product semantics, it is because the reality of hedonic shopping is emotional and cultural based on product semantics. On the one hand, the product design team carefully explains the “fashion,” “cute,” “safe,” “warm,” “elegant,” “simple,” and other semantic attributes, merchants strive to enlarge semantic symbols through the atmosphere of the store, product landscaping, etc. in order to stimulate consumers' desire to buy; on the other hand, consumers will comprehensively evaluate the purchase risk before buying, and rationally choose the purchase decision with the least possibility of regret. However, they often “buy unnecessary products,” thus forming an interesting game of “temptation” and “regret.” Based on the discussion above, the conceptual model of this study can be built as shown in Figure 1.

## Research Hypotheses

### Product Semantic Perception and Online Impulse Purchase Intention

According to Verganti (2003), product semantics can be understood as “a set of symbols, symbols and images that can express emotions and symbolic values.” It is believed that product semantics could be defined as meaning, that is, consumers' interpretation of Internet product symbols, which determines the cultural, emotional, and symbolic attributes of products (Chen and Chen, 2016). Product semantics are different from product attributes and the latter refers to “features that enable consumers to meet certain needs through purchase.” It mainly includes observably functional characteristics such as specifications and prices, and features composed of product performance and technical characteristics. It is argued that many retailers are trying their best for ways to “fine-tune” their product characteristics while maintaining variety and differentiation (Progressive Grocer, 2011). Kacen et al. (2012) further pointed out that hedonic, ready-to-use, low-priced, and specially displayed products are those with a higher likelihood of being bought on impulse. Thus, these findings can help retailers to make strategic decisions about which products to put away or remove from store shelves in order to increase sales. However, research investigating the link between product semantic perception and impulse buying remain scant in the literature.

The connotative meaning of product semantic perception emphasizes the spiritual and cultural attributes of the product that can be perceived by consumers (Alwi et al., 2014). Product semantics are the emotional and value propositions such as “fashion,” “cute,” “safe,” “warm,” “elegant,” “simple,” and so on which can help identify different products. Combined with the theory of product hierarchy, this study defines product semantics as a combination of symbols attached to the core product to meet the psychological needs of online consumers such as culture, emotions, and symbolic meaning.

Scholars from different fields have given different explanations on the mechanism of the influence of product semantics on purchasing decisions. Under the influence of consumerism, consumers' attitude of “indirect utility” had a profound impact on consumption behavior, and symbols became the manifestation of personality. The symbolic value of products was often regarded as the main value of products. In fact, due to the changes of the internal and external environment, online consumers purchase Internet products not only to meet functional needs, but also tend to seek the cultural, emotional and symbolic meanings contained in the products themselves, which determines that product semantics help to upgrade the cultural consumption of products (Li et al., 2018). Thus, it is highly possible that product semantics perception of the consumers lead to impulse buying.

Other scholars have interpreted it from the perspective of engineering psychology, and verified that the implicit attributes of products such as hedonism, beauty, and culture can effectively evoke consumers' emotions, and then influence purchase decisions (Chattaraman et al., 2016). It can be seen that non-functional requirements have become an important influencing factor for customers' purchasing decisions in a post-modern society centered on consumption. Compared to practical shopping which aims to obtain the direct utility of products and services, online shopping which pursues for more hedonism is mainly concerned with spiritual and cultural needs such as satisfaction, excitement, and escape in the shopping process, that is, the indirect utility attached to product symbols (Gao and Shen, 2016).

As well as combined with the connotation of the mental account discussed in the previous section, it can be seen that the semantic perception of the product changes the consumers' mental account, which in turn stimulates irrational impulse consumption of Internet products in pursuit of emotional satisfaction. Thus, the hypothesis is proposed accordingly:

H1: Product semantic perception positively affects online impulse purchase intention.

### Product Semantic Perception and Expected Inaction Regret

Given that the functional homogenization of product is growing, the semantic design of the product which is based on the cultural, emotional, and symbolic meaning attached to the Internet product is more and more appreciated by online businesses. According to Buttner et al. (2014), non-functional needs such as pleasure, entertainment, social interaction, killing time, and self-satisfaction in the shopping process are increasingly sought after by online consumers, so the aesthetic design and semantic cultural design of products are increasingly highlighted.

When the value of product semantic perception is higher, online consumers will have a higher sense of belonging, identity, and efficacy, which will result in a higher sense of psychological ownership. Yeung (2012) believed that psychological ownership will cause deviation in product evaluation. Once people have psychological ownership of something, on the one hand, they will improve the perceived value and emotional attachment of possession; on the other hand, they will increase their sensitivity and pain of losing possession. In addition, Jussila et al. (2015) emphasized that consumers will seek a match between their own value and the product culture when purchasing, which improves the individual's perception of psychological ownership and positively affects customer satisfaction, loyalty, and willingness to pay significantly. Therefore, product semantic perception aroused by well-designed semantic attributes determines the spiritual and cultural needs of hedonic purchasing for Internet products, which affects the psychological account of consumers, and causes consumers to regret not taking expected actions: “if they don't buy, they may have expected losses.” The higher the semantic perceived value of the product, the stronger the anxiety of “buy now or regret later” of online consumers. Hence, the following hypothesis is proposed:

H2: Product semantic perception value positively affects expected inaction regrets.

### Expected Inaction Regrets and Impulse Buying for Internet Products

The current research on expected regret and purchase behavior has not carried out further subdivided research on expected regret. Expected inaction regrets are the same as expected regrets and both of them are subjective imaginations made by online consumers based on expected loss aversion, regret aversion, or uncertainty aversion, with the purpose of minimizing regret decisions with expected future results (Luce, 1998). Expected inaction regret is related to Roses's (1994) counterfactual thinking theory, which infers how the opposite situation may affect the current results by imagining the opposite situation, that is, what would the possible consequences of failing to do so be. In order to reduce the possible losses caused by expected inaction regret, online consumers need to take counterfactual thinking into account and take relevant actions, which determines that there is a positive correlation between their expected inaction and impulse purchase for Internet products.

In fact, this relationship has been partly supported by some empirical studies. Research carried out by Yin and Yu (2009) showed that the direction of expected regret can directly affect impulsive purchases. More direct evidence comes from Chen et al. (2011)'s research on China's real estate market. Expected inaction regret intensifies the anxiety and dissatisfaction in the context of the non-purchase of real estate. It is believed that online consumers usually resort to impulse buying in order to reduce the negative emotions of anxiety. It is easy to understand that if the Internet products can give consumers higher semantic perceived value, it will increase the risk of loss of expected inaction, while counterfactual thinking will prompt online consumers to undertake purchasing behaviors to reduce expected losses. Thus, Hypothesis 3 is proposed as follows:

H3: Expected inaction regret positively affects impulse buying.

### Mediating Effect of Expected Inaction Regret

The classic “stimulus-organism-response” (SOR) model can be used to explain the mediating effect of expected inaction regret between product semantic perception and impulse purchases for Internet products. The SOR model, proposed by Woodworth (1928), is commonly used as a theory basis to investigate online impulse buying because this framework has traditionally provided the foundation for consumer behavior studies (Chan et al., 2017; Zheng et al., 2019). According to studies on the context of impulse buying for Internet products, this framework is continuously tested on the relationships between product semantic perception stimuli, customers' expected inaction regret organisms, and online impulse buying behavioral responses.

Product semantics can explain and express the spiritual and cultural attributes contained in products to online consumers through product function combination, form, and appearance design, and various symbols such as text and diagrams. Businesses can also further stimulate consumption desire of Internet products through the creation of an online shopping environment and the interaction of making good friends. This kind of stimulation can enhance expected inaction regret, which further affects the attitude of possession, attitude of belonging, and online purchase behavior. The mediating mechanism of expected inaction regret has been supported by some related studies. For example, Beltagui et al. (2012) found that the aesthetic value of beauty and goodness in products, as well as environmentally friendly values, can both stimulate consumers' pro-social or personal motivation and ultimately increase their desire for consumption experience. In addition, the mediating mechanism of expected inaction regret can also be further explained by the classical effect level model (Lavidge and Steiner, 1961). According to the effect hierarchy model of “cognition-emotion-meaning,” product semantic perception is first the subjective cognition of consumers (the first layer). This kind of cognition stimulates consumers' anxiety emotion (the intermediary layer); and finally promotes consumers to generate “if not bought, there may be a loss” intention (the third layer), and induces consumers to make online impulsive purchases. Thus, the following hypothesis is brought forward:

H4: Expected inaction regret has a mediating mechanism between semantic perception of the product and impulse buying for Internet products.

### The Moderating Effect of Self-Control

Self-control is a conscious self-regulation process. It is an individual's adaptive response ability to suppress emotions, desires, ideas, and behaviors, and to achieve individual expectations and social expectations (Gailliot et al., 2007). Online impulsive purchase is the performance of one-sided cognition, reckless decision-making, emotional indulgence, and lack of self-control behavior. Wang and Yao (2018) believed that according to the “desire-willpower” model of impulsive behavior, consumers with impulsive desire may not really make impulsive purchases, and consumers will have corresponding self-control measures. Only when the desire exceeds willpower will online consumers' self-control fail and they make impulse purchases for Internet products. Therefore, even if online consumers have a strong anticipation of inaction regret, self-control will affect impulse purchase behavior.

According to Sultan et al. (2012), self-control is a selective tendency formed between consumers' emotional preference and impulsive consumption. Compared with low self-control ability, online consumers with high self-control are more likely to resist external temptation (Lai et al., 2016). In addition, Luo (2018) took cosmetics consumption in the e-commerce environment as an example and found that consumer self-control played a negative regulatory role between consumer sentiment and impulsive purchases. Therefore, the self-control ability of online consumers can regulate the relationship between expected inaction regret and their impulsive purchases for Internet products, and the stronger the self-control ability is, the more able they are to resist the negative perception of loss associated with expected inaction regret, thus weakening the occurrence of online impulsive purchase behavior. Hence Hypothesis 5 is proposed as follows:

H5: Self-control negatively moderates the relationship between expected inaction regret and impulse buying for Internet products.

## Research Design and Process

### Measurement of Variables

To confirm and ensure the validity of the measurement instrument, back translation of the questionnaire for this study was done. The English version was firstly translated to Chinese and then the Chinese questionnaire was back translated to English by another individual. The two English versions of the questionnaire were compared to ensure their equivalence. On this basis, the applicability of the scale was tested through the researchers' collective research and small-scale investigation, three Internet marketing experts and five experienced online consumers were involved in the pretest and the items were adjusted in terms of wording and rhetoric, so as to finally complete the questionnaire design. All variables were measured with a seven-points Likert scale ranging from “1-strongly disagree” to “7-strongly agree.” Finally, a total of 19 measurement items including the semantic perception of product (stimulus), expected inaction regret (organism), online impulse buying (response), and self-control (moderator) from the SOR model are described and listed in Supplementary Table 1 based on the literature and discussions above.

### Research Design

In this paper, the primary data were obtained by the experimental method. The experiment was carried out from March 1 to March 22, 2019, and the subjects were office staff of eight garment manufacturing enterprises in Changzhou and Nanjing of Jiangsu Province through convenience sampling. In order to get close to the actual online shopping scene, a mini humidifier was selected as the experimental material from the famous B2C e-commerce platform 360Buy. There were three reasons for this selection: firstly, the product is suitable for consumers' real needs, for example, many offices use humidifiers because of the dry environment caused by central air conditioning in those offices; secondly, the product price is not high, consumers have purchasing power and the possibility to buy; and thirdly, the semantic design of products has a large space for innovation, which is convenient to distinguish the variable measurement level. To avoid interference to the experiment caused by function, price, and other factors, the experimental materials were all mini humidifiers with a single function, consistent price, convenient use, and the same brand and after-sales service.

The experiment was conducted separately in the company in groups A and B. The unit price of the humidifier in experiment A was 129 yuan, and its appearance was like that of a household electric rice cooker. In the experiment, simple pictures, sources, and a brief description were provided on the website, and the product semantics were relatively simple. The unit price of the humidifier for group B was also 129 yuan, and had the same functional description. However, the humidifier looked just like the head of a cute pig on the website, with the water vapor curling out from the two large nostrils. Simultaneously, the following pictures and text semantics were shown on the website as well: firstly, warm, peaceful, elegant, and other fragmentary text descriptions were provided to the subjects of group B. Secondly, a picture showing a successful white-collar worker beside the humidifier drinking Starbucks coffee was given and shown on the website; thirdly, a picture demonstrating that the humidifier was properly used to create a simple and delicate environment was shown to the online subjects. And lastly, the values of “work and life” were also promoted to the subjects. The experimental design highlights the consistency of Internet product functions and the differences in product semantic perception. It emphasizes the emotional and cultural attributes of the humidifier and maps online consumers' personalities, tastes, images, identities, and values through semantic symbols. The subjects were placed in comfortable chairs and asked to complete the questionnaire independently. In order to thank the subjects for their cooperation and support, they were given small gifts such as nail clippers before the experiment. Then the experiment was launched by asking the subjects to browse the product on the webpage and then the paper questionnaires were distributed for the subjects to fill in and collected on the spot.

### Data Collection

The subjects were randomly assigned to group A and group B and filled in the questionnaire after watching the introduction materials demonstrated by a projector. The first part of the questionnaire consisting of three questions was to help determine whether the subject had entered the experimental situation, if they failed to answer these questions correctly, their questionnaires were deleted according to the method offered by Eisend (2008). The three questions were: ① What do you think of Internet products?; ② Do you need this product?; ③ If you already have the product, would you like to buy it as a gift for others? Then, their answers were discarded if the answer to question ① was not a humidifier, and the questionnaire was also invalid if both options of questions ② and ③ were <2. In addition, questionnaires with more than three missing items, irregular filling, and too low choice discrimination were all taken as invalid samples.

A total of 680 office workers participated in the test and filled in the questionnaire. There were 57 invalid samples which were removed, and 623 valid questionnaires were finally collected. The descriptive analysis of the samples is shown in Table 1. A total of 342 respondents were from Nanjing, occupying 54.9% of the sample while 281 respondents were from Changzhou, occupying 45.1% of the total sample. Among the samples, 205 respondents were under 30 years old, accounting for 32.9% and there were 170 aged 31–40, accounting for 27.3%. In addition, 158 respondents were aged 41–50, accounting for 25.3% while 90 respondents were over the age of 51, accounting for 14.5%. For different genders, 338 respondents were men, accounting for 54.2% of the total sample while 285 respondents were women, accounting for the other 45.8%. In terms of the education level of the sample, 208 respondents had high school or technical secondary school degree, 300 respondents had a college degree, and 115 respondents had a bachelor's degree or master's degree; different educational level groups accounted for 33.3, 48.2, and 18.5% of the total sample, respectively. As for the involved groups in terms of different product semantic perception, 283 respondents were in group A while group B had 340 respondents.

**Table 1:** Descriptive analysis of samples.

		**City**		**Gender**		**Education level**		
		**Nanjing**	**Changzhou**	**M**	**F**	**High school**	**College**	**Higher than bachelor's degree**
Age	<30	112	93	120	85	70	95	40
	31–40 years old	90	80	98	73	58	78	35
	41–50 years old	90	68	73	85	50	77	30
	More than 51	50	40	47	42	30	50	10

## Data Analysis

### Common Method Variance Analysis

The autocorrelation of the sample may lead to common method biases, which could be caused by the artificial co-variability between predictors and criteria due to the same data sources or raters, the same measurement environment, the project context, and the characteristics of the project itself. Therefore, two ways were used to check for common method variance. One was to check the correlation coefficient between the four variables, such as product semantic perception. It can be seen from Table 2 that the maximum correlation coefficient between variables was 0.471, thus there was no strong correlation. The Harman single factor test method was the second method. A one-time exploratory factor analysis was performed with the four variables of product semantic perception. It was found that the interpretation rate of the first principal component was 27.058% when the eigenvalue was >1 and no rotation was done, accounting for less than the critical value of 50%, which indicated that homologous deviation would not have a substantial impact on the data validity analysis.

**Table 2:** Reliability and validity.

**Variables**	**Measuring item**	**Factor loading**	**Cronbach's α**	**CR**	**AVE**	**Model fitness index**
Product semantic perception	PM1	0.808	0.904	0.948	0.821	CMIN/DF = 1.606
	PM2	0.776				RMSEA = 0.049
	PM3	0.815				GFI = 0.994
	PM4	0.975				CIF = 0.998
Anticipated regret	AR1	0.684	0.748	0.831	0.552	CMIN/DF = 2.370
	AR2	0.619				RMSEA = 0.074
	AR3	0.679				GFI = 0.972
	AR4	0.631				CIF = 0.978
Impulse buying	IB1	0.696	0.827	0.893	0.676	CMIN/DF = 2.210
	IB2	0.774				RMSEA = 0.070
	IB3	0.744				GFI = 0.991
	IB4	0.740				CIF = 0.993
Self-control	SC1	0.788	0.888	0.935	0.741	CMIN/DF = 2.392
	SC2	0.798				RMSEA = 0.075
	SC3	0.777				GFI = 0.981
	SC4	0.770				CIF = 0.989
	SC5	0.792				

### Reliability and Validity Analysis

Cronbach's coefficient was used to test the internal reliability of variables, and AMOS software was used to conduct confirmatory factor analysis. The test results are shown in Table 1. The reliability coefficients of the four variables were all >0.7, indicating that the scale had a high internal consistency. The factor load values were all above 0.6, implying a high correlation between measurement items and latent variables. The combined reliability CR values were all >0.6, suggesting that there was a large internal consistency between the observed variables and the corresponding latent variables. The mean variance extraction value AVE of each latent variable was >0.5, indicating that the observed variable can reflect the internal characteristics of the common latent variable and the aggregation validity was high.

Table 2 also gives the four main indicators of confirmatory factor analysis. In comparison with the standard of model fitness statistics, although some of the RMSEA value fitness evaluation indexes did not reach the perfect standard (<0.05), they were close to the reasonable range of model fitness (<0.08), suggesting that the variable measurement had a high validity.

### Descriptive Statistics and Correlation Analysis of Variables

The analysis results of mean, standard deviation, and correlation coefficient of variables are shown in Table 2. As can be seen from Table 3, there was no significant correlation between age, education background, and semantic perception of products, but there was a significant correlation between gender and semantic perception of products (γ = 0.280**) and expected inaction regret (γ = 0.172**). There was a significant positive correlation between product semantic perception, expected inaction regret, and impulse purchase intention. There was a significantly negative correlation between self-control and impulse purchase intention for Internet products (γ = −0.429**). According to the relevant research of Yu et al. (2016), if the correlation coefficient between variables is all <0.7, it indicates that the concept distinction of variables is reasonable and there is no collinearity threat, so the four variables can be well-distinguished and there was no collinearity, which lays a foundation for further hypothesis testing.

**Table 3:** Descriptive statistics and correlation analysis of variables.

	**M**	**SD**	**Age**	**Gender**	**Education**	**PM**	**AR**	**IB**
Age	3.210	1.058						
Gender	1.460	0.499	0.074					
Education	1.850	0.706	−0.022	0.057				
PM	4.524	1.078	0.022	0.148^*^	−0.039			
AR	4.456	0.823	0.021	0.172^**^	0.056	0.471^**^		
IB	4.717	0.693	−0.071	0.094	0.128	0.212^**^	0.219^**^	
SC	4.124	0.941	−0.018	0.030	−0.068	0.001	0.060	−0.429^**^

* *indicates p < 0.1*.

** *Indicates p < 0.01, double-tailed test*.

*PM stands for product semantic perception, AR for expected inaction regret, IB for impulse purchase intention, and SC for self-control*.

### Hypotheses Testing

The measurement items of four variables were averaged and decentralized. Hierarchical regression analysis was used to test the relationship between the variables, and the results are shown in Table 4. Model 1 represents the influence of control variables such as age and product semantic perception on expected inaction regret. The equation as a whole passed the significance test with a determination coefficient of 0.237. Product semantic perception significantly affected expected inaction regret (β = 0.350***), and H2 was assumed to pass the test. Model 2 tested the influence of semantic perception of products on impulse purchase intention. F statistic showed that the equation as a whole passed the significance test with a determination coefficient of 0.173, and the semantic perception of products positively affected expected inaction regret significantly (β = 0.135***), and H1 passed the test. Both Model 1 and Model 2 showed that there were significant differences between gender and the explained variables. Further, independent sample T analysis was used to test the difference of gender in expected inaction regret, and significant differences were found (T = −4.592, *p* < 0.001) among men (*M* = 4.539, *SD* = 0.841) and women (*M* = 5.004, *SD* = 0.738). Similarly, Model 3 also showed that expected inaction regret positively affected online impulse purchase intention (β = 0.172***) significantly, thus H3 passed the test.

**Table 4:** Regression analysis on hypothesis testing.

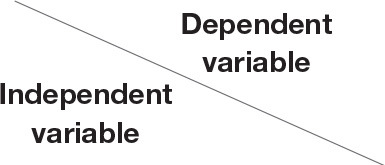	**Model 1**	**Model 2**	**Model 3**	**Model 4**	**Model 5**	**Model 6**	**Model 7**
	**AR**	**IB**	**IB**	**IB**	**IB**	**IB**	**IB**
Constant term	−0.402	−0.198	−0.157	−0.152	−0.204	−0.100	−0.032
Age	0.004	−0.051	−0.051	−0.052	−0.056	−0.057	−0.065
Gender	0.165^*^	0.084	0.080	0.066	0.149	0.096	0.082
Education	0.080	0.129	0.109	0.119	0.089	0.078	0.068
PM	0.350^***^	0.135^***^		0.095^***^			
AR			0.172^***^	0.115^***^		0.194^***^	0.190^***^
SC					−0.315^***^	−0.325^***^	−0.336^***^
AR × SC							−0.137^**^
R2	0.237	0.173	0.170	0.327	0.211	0.263	0.289
F	18.981^***^	4.770^***^	4.589^***^	4.612^***^	16.333^***^	17.313^***^	16.401^***^
VR^2^							0.026
D.W.	2.057	1.958	1.980	1.969	1.960	1.977	2.004

*** *Indicates p < 0.001*,

** *indicates p < 0.01*,

* *indicates p < 0.1*.

*PM stands for product semantic perception, AR for expected inaction regret, IB for impulse purchase intention, and SC for self-control*.

Referring to the test of mediating effect, the first step was to test the independent variable product semantic perception through Model 1, which positively affected the mediating variable expected inaction regret significantly (β = 0.350**). The second step was to test the semantic perception of independent variable products through Model 2, which positively affected the dependent variable online impulse purchase intention significantly (β = 0.135***). The third step was to test (through Model 3) that the mediating variable expected inaction regret positively affected the dependent variable online impulse purchase intention significantly (β = 0.172***). Based on the first three steps, analysis of Model 4 found that the two variables of semantic perception, anticipated inaction regret, and equations all achieved significance level. In addition, expected inaction regret played a partial intermediary role between semantic perception of products and impulse purchase intention; and the level of mediating effect was 0.350 × 0.115 = 0.040, which was the difference between the total effect (β = 0.135***) and the direct effect (β = 0.095), thus H4 passed the test.

**Figure 2:**
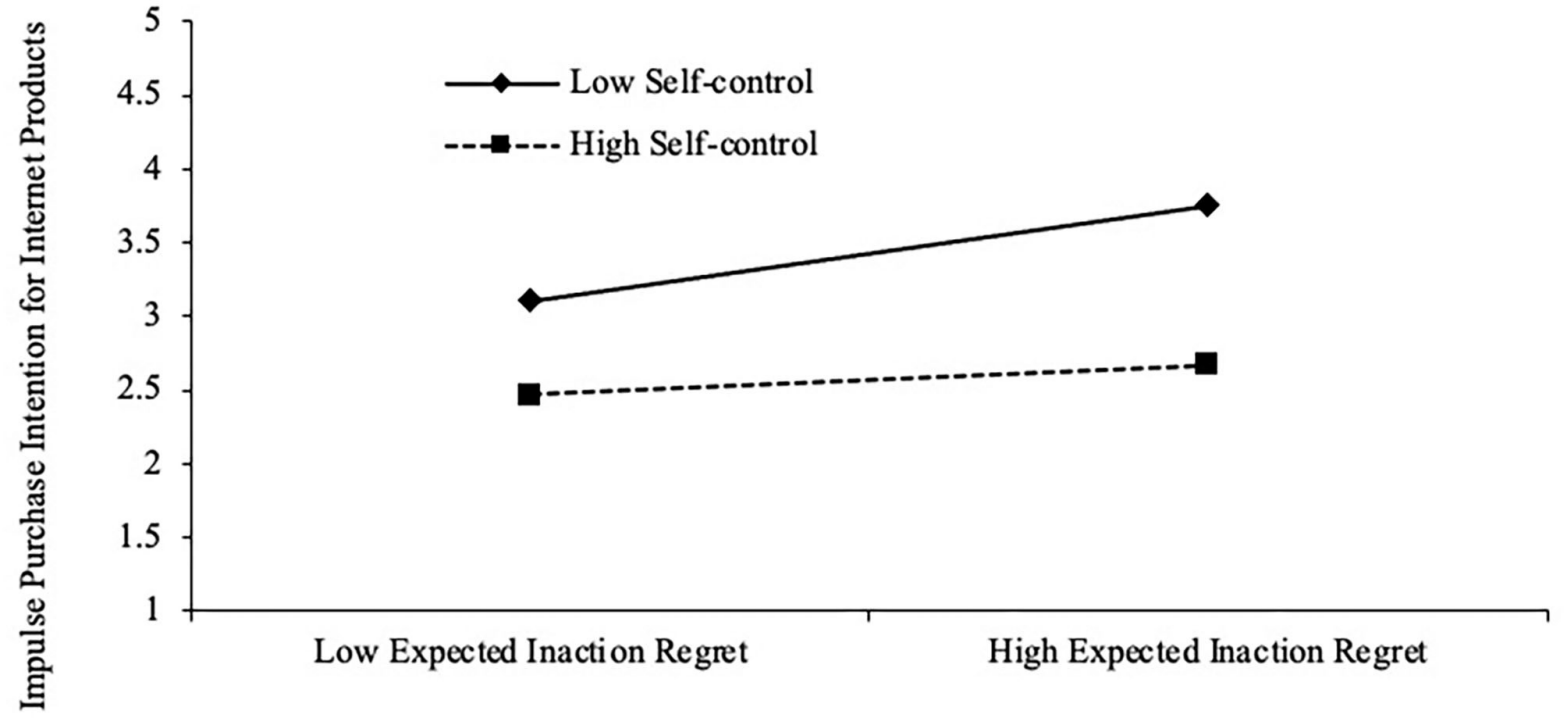
The regulating effect of self-control.

Model 5 shows that self-control had a negative effect on impulse purchase intention for Internet products (β = −0.315***), which provides a possibility for self-control to exert a moderating effect. Model 6 also included expected inaction regret and self-control into the regression model. It was found that the two variables and the equation passed the significance test, with a determination coefficient of 0.263. Model 7 considers the “interaction between expected inaction regret and self-control,” the results show that expected inaction regret affected the impulsive purchase intention (β = 0.190***), and self-control still negatively impacted online impulse purchase intention (β = −0.336***). The interaction between expected inaction regret and self-control negatively affected impulsive consumption (β = −0.137**), indicating that the relationship between expected inaction regret and impulse purchase intention for Internet products was a function of self-control with a moderating effect of −0.137. Therefore, H_5_ passed the test. A simple slope estimation was used to plot the moderating effect, as shown in Figure 2. Self-control weakened the positive effect of expected inaction regret on impulse purchase intention, that is, compared with low self-control, when consumers had high self-control ability, the positive relationship between expected inaction regret and impulse purchase intention for Internet products was weaker.

## Conclusion

### Findings

Drawing upon product semantic perception as an overarching theoretical framework, this study identifies and conceptualizes a salient purchasing decision mode, and examines how product semantic perception influences impulse buying of consumers for Internet products. It is found that product semantic perception has significant influences on impulse buying through the mediation effects of expected inaction regret. Furthermore, self-control significantly moderates the influences of the expected inaction regret mechanisms on online impulse buying. Overall, our empirical results support all of the proposed hypotheses and generate several new research findings. We summarize the key research findings of this study as follows.

First, this study establishes strong links among product semantic perception, expected inaction regret, and impulse buying for Internet products in the context, responding to the call for more empirical studies to investigate the influences of product semantic perception in new research contexts (Diagne et al., 2016; Eickhoff et al., 2020). Our research findings confirm the significant role of higher product semantic perception in stimulating a higher level of “loss” perception and anxiety after the consumption experience (Camille et al., 2004; Sandberg et al., 2016), leading to a higher extent of expected inaction regret. In addition, the semantic perception of products has a significant positive impact on the expectation of inaction and regret, also indicating that the “symbol combination attached to the core products” can meet the psychological needs of online consumers such as culture, emotion and symbolic meaning, and create customer value just like the use function of tangible products. Besides, women are more likely to have higher levels of expected inaction regret than men, which is consistent with the extant literature (Dittmar et al., 1996; Lucas and Koff, 2014). Furthermore, expected inaction regret positively affects impulse purchase for Internet products, indicating that online consumers will still make impulse purchase decisions based on regret minimization even in the face of the semantic “non-use function” demand of products.

Second, this study obtains new research findings by uncovering the mediation mechanism of expected inaction regret between product semantic perception and impulse buying. As reported in the mediation test with the results from regression analysis, expected inaction regret plays a partial mediating role between the semantic perception of products and online impulse purchase intention. Previous research findings suggested that there were consequences of impulse buying such as regret and unnecessary spending, thus strategies like post-purchase communication, seeking social support, and so on have been proposed to cope with expected action regret (Yi and Baumgartner, 2011; Chang and Tseng, 2014). However, few studies have examined the role of expected inaction regret, thus our results extend the previous literature by identifying the mediation effects of expected inaction regret between product semantic perception and online impulse buying. Thus, e-business platforms can more effectively implement product semantic designs, enhancing consumers' sense of pleasure, desire, and impulse, implying the possible losses that might be brought about by expected inaction, and facilitating their online purchases.

Third, this study provides empirical support for previous theoretical studies by responding to their calls for investigating the moderating effects of self-control in different consumer behavioral contexts (Kchaou and Amara, 2014; Dhandra, 2020). In particular, our study incorporates self-control as an anticipated factors, and uncovers its moderating effects on impulse buying mechanisms. Our research findings suggest that self-control negatively moderates the relationship between expected inaction regret and impulse purchases for Internet products, indicating that online consumers will make a comparison between “desire and willpower” before impulse purchases, and self-control weakens the positive effect of expected inaction regret on online impulse purchases. This confirms the viewpoint that gratification and desire for the product can override self-control of consumers which leads to impulse buying (Hoch and Lowenstein, 1991). Therefore, it is interesting and necessary to study the influence of consumerism on the semantic symbols of products to stimulate the purchasing psychology of expected inaction and induce online impulse buying of consumers.

### Theoretical Contributions

Our study makes three contributions to the extant literature. First, we establish a theoretical link between product semantic perception and online impulse buying. While previous studies mostly considered psychological factors (Rook and Gardner, 1993; Beatty and Ferrell, 1998; Amos et al., 2014; Bandyopadhyay, 2016), lack of control (Youn and Faber, 2000; Parboteeah et al., 2009; Amos et al., 2014), personality traits (Verplanken and Herabadi, 2001; Chan et al., 2017; Satyavani and Chalam, 2018), demographic characteristics (Dittmar et al., 1996; Wood, 1998; Silvera et al., 2008), environment of retailers, product mix design, and promotion design (Zhou and Wong, 2004; Kacen et al., 2012; Mohan et al., 2013), the role of product semantics in triggering consumers' impulse buying for Internet products remains largely unexplored. To our knowledge, this is one of the first studies that brings product semantic perception to the context of impulse buying. Based on the impulse buying theory and mental accounts theory, our study examines the relationship between product semantic perception and online impulse buying. These research findings can enrich our understanding of impulse buying from a product semantics theoretical perspective.

Second, our study uncovers the mediation effects between expected inaction regret and online impulse buying. Previous studies, though still very a few, considered expected action regret as a construct and investigated the influence of this kind of regret on consumers' online behaviors from a psychologically theoretical perspective (Yi and Baumgartner, 2011; Chang and Tseng, 2014). While in increasing online shopping festivals like “Double Eleven,” consumers engage in enjoyment and pleasure and sustain possible losses with inaction regret. In particular, we find that expected inaction regret plays a partial mediating role between the semantic perception of products and online impulse purchase intention. The research findings could interpret the role of expected inaction regret in the context of the rise of the e-commerce era from an affective and psychological theoretical perspective.

Third, our study reveals the moderating effects of self-control on the relationship between expected inaction regret and impulse purchases for Internet products. While prior studies have delved into self-control in the impulse buying context (Parboteeah et al., 2009; Amos et al., 2014; Kchaou and Amara, 2014; Dhandra, 2020), little attention has been paid to the role of self-control concerned with expected inaction regret. Therefore, our empirical results can further enhance our understanding regarding the anticipated effects of self-control in the context of online impulse buying.

### Practical Implications

The findings of this study can provide practical guidelines for business developers and operators of online purchase platforms.

First, platform developers and operators need to recognize the importance of the semantic symbol design of product “non-use function” in stimulating impulse buying. On one hand, platform developers and operators can implement semantic symbol design to stimulate consumers to impulsively buy Internet products. Under the wave of consumerism, online consumers pay much attention to the comprehensive experience of emotion in the process of Internet products purchases, and the pragmatic design of products finds it difficult to meet their spiritual and cultural needs. Only through the breakthrough innovation of product semantics can e-commerce enterprises better meet demand and lead a consumption trend. Design becomes the third-dimension innovation driving force besides technology and marketing as design endows products with culture, emotion, and symbolic meaning, and can meet the spiritual and cultural needs of the hedonic consumption process for Internet products. It is worth noting that online platform developers should always remind themselves that semantic design of products cannot be too complicated since many consumers have little time or energy to understand the intricate information transferred to them by the highly competitive online market. Thus, product development requires the design and research team to interpret product symbols well and match them to their potential consumers. Moreover, e-business operators and marketers should recognize that the semantic attributes contained in the product itself with high expected inaction regret can help enhance purchase experience and induce impulse buying.

Second, platform developers and operators should take self-control into consideration when developing product semantic perception, and design the most attractive non-functional features to increase consumers' impulse buying through the Internet. Chinese online consumers' loss aversion and self-control make them rationally control their emotions or desires in the face of the temptation of semantic symbols of Internet products, so as to avoid the “regret” result of substantial purchase losses. Therefore, it is important to stimulate the visual, auditory, tactile, and other senses of consumers, so as to confront the negative influence of self-control and to improve their perception of possible losses caused by expected inaction and stimulate online purchase desire.

### Limitations and Research Directions

While our study provides salient theoretical and practical contributions, there are several limitations that may point to future research directions. First, the sample of our questionnaire survey is relative limited. To generalize the research findings of our study, future research can collect data from a wider sample and may be from different countries, thereby investigating if the product semantic perception mechanisms on impulse buying are contingent upon different cultures. Second, our study considers impulse buying as an overall second-order construct. In order to obtain more interesting research results, future research should delve into the specific effects of product semantic perception on different types of impulse buying. Thirdly, our data sample paid little attention to the demographic characteristics of consumers. Further research can collect data from different age groups, occupation groups, and so on to see whether there are differences in findings. Last but not least, future studies can employ a more rigorous neurophysiological design to assess actual impulse buying behaviors, such as measuring blood flow, muscle activation, and brain activity, to avoid the potential common method bias.

## Data Availability Statement

The raw data supporting the conclusions of this article will be made available by the authors, without undue reservation.

## Ethics Statement

Ethical review and approval was not required for the study on human participants in accordance with the local legislation and institutional requirements. Written informed consent for participation was not required for this study in accordance with the national legislation and the institutional requirements.

## Author Contributions

RC contributed to the conceptualization, methodology, supervision, project administration, and validation of this paper. XH was responsible for writing—review and editing and formal analysis. Both authors contributed to the article and approved the submitted version.

## Conflict of Interest

The authors declare that the research was conducted in the absence of any commercial or financial relationships that could be construed as a potential conflict of interest.

## Publisher's Note

All claims expressed in this article are solely those of the authors and do not necessarily represent those of their affiliated organizations, or those of the publisher, the editors and the reviewers. Any product that may be evaluated in this article, or claim that may be made by its manufacturer, is not guaranteed or endorsed by the publisher.
