# Supplementary material for: Does Product Semantics Matter in Stimulating Impulse Buying Behavior for Internet Products?
Source: Front Psychol. 2021 Aug 23;12:676086. doi: 10.3389/fpsyg.2021.676086 (PMC8419358; doi:10.3389/fpsyg.2021.676086)
Supplement: Supplementary file 1 [file Table_1.docx]

Supplementary Material

## Supplementary Tables

Table S1 Measurement of Variables

| SOR Model | Variables | Measurement Items | Sources |
| --- | --- | --- | --- |
| Stimulus | Product semantic perception | 1. Conforming to the personality or taste of online consumers 2. Help to improve the image of online consumers 3. Help to distinguish online consumers from those around them 4. Be able to symbolize and express the identity or achievement of online consumers | Homburg et al.(2015); Lai et al.(2016) |
| Organism | Expected inaction regret | If I do not buy,   1. I will feel guilty 2. I feel self-accusation 3. I would be sorry 4. I would be disappointed | Chen (2007); Hao & Zeng (2017) |
| Response | Online impulse buying | When I see the internet products,   1. Own the commodity immediately 2. I have the desire to buy this product 3. I thought the product was what I wanted 4. Although it was not the product I planned to buy before, I really want to buy it now | Beatty & Ferrell (1998); Luo (2018) |
| Moderator | Self-control | 1. Usually I know why I am happy 2. I can clearly recognize and experience my emotional state 3. When I am sad I always have a way to make myself happy 4. I know what causes me pain 5. Usually I can accurately express my emotions. | Gross & John (2003) |
